# Maladaptive Pulmonary Vascular Responses to Chronic Sustained and Chronic Intermittent Hypoxia in Rat

**DOI:** 10.3390/antiox11010054

**Published:** 2021-12-27

**Authors:** Jesus Prieto-Lloret, Elena Olea, Ana Gordillo-Cano, Inmaculada Docio, Ana Obeso, Angela Gomez-Niño, Philip I. Aaronson, Asuncion Rocher

**Affiliations:** 1 Departamento de Bioquimica y Biologia Molecular y Fisiologia, Facultad de Medicina, Universidad de Valladolid, 47005 Valladolid, Spain; jesus.prieto@uva.es (J.P.-L.); anagorca@ibgm.uva.es (A.G.-C.); inmaculada.docio@uva.es (I.D.); aobeso@ibgm.uva.es (A.O.); 2 Unidad de Excelencia, Instituto de Biología y Genética Molecular (IBGM), Universidad de Valladolid-CSIC, 47005 Valladolid, Spain; elena.olea@uva.es (E.O.); angeles.gomez.nino@uva.es (A.G.-N.); 3 Departamento de Enfermeria, Facultad de Enfermeria, Universidad de Valladolid, 47005 Valladolid, Spain; 4 Departamento de Biologia Celular, Histologia y Farmacologia, Facultad de Medicina, Universidad de Valladolid, 47005 Valladolid, Spain; 5 Department of Inflammation Biology, School of Immunology and Microbial Sciences, Faculty of Life Sciences and Medicine, King’s College, London SE1 1UL, UK; philip.aaronson@kcl.ac.uk

**Keywords:** chronic intermittent hypoxia, systemic and pulmonary hypertension, obstructive sleep apnea, endothelium dysfunction, nitric oxide, methylated arginines

## Abstract

Chronic sustained hypoxia (CSH), as found in individuals living at a high altitude or in patients suffering respiratory disorders, initiates physiological adaptations such as carotid body stimulation to maintain oxygen levels, but has deleterious effects such as pulmonary hypertension (PH). Obstructive sleep apnea (OSA), a respiratory disorder of increasing prevalence, is characterized by a situation of chronic intermittent hypoxia (CIH). OSA is associated with the development of systemic hypertension and cardiovascular pathologies, due to carotid body and sympathetic overactivation. There is growing evidence that CIH can also compromise the pulmonary circulation, causing pulmonary hypertension in OSA patients and animal models. The aim of this work was to compare hemodynamics, vascular contractility, and L-arginine-NO metabolism in two models of PH in rats, associated with CSH and CIH exposure. We demonstrate that whereas CSH and CIH cause several common effects such as an increased hematocrit, weight loss, and an increase in pulmonary artery pressure (PAP), compared to CIH, CSH seems to have more of an effect on the pulmonary circulation, whereas the effects of CIH are apparently more targeted on the systemic circulation. The results suggest that the endothelial dysfunction evident in pulmonary arteries with both hypoxia protocols are not due to an increase in methylated arginines in these arteries, although an increase in plasma SDMA could contribute to the apparent loss of basal NO-dependent vasodilation and, therefore, the increase in PAP that results from CIH.

## 1. Introduction

Pulmonary and systemic hypoxemia, sustained or intermittent over time, triggers homeostatic responses that tend to restore normal levels of oxygen, as well as pathological processes caused by adverse tissue adjustments. Chronic sustained hypoxia (CSH), resulting from habitation at a high altitude or from respiratory disorders (i.e., COPD), initiates physiological adaptations that include the activation of carotid body (CB), eliciting hyperventilation; an increase in red blood cell production, which improves the O_2_ carrying capacity; angiogenesis to facilitate the blood flow and oxygen transport to the tissues; and cell metabolic re-programming, which reduces O_2_ consumption [1,2]. However, CSH may also have deleterious effects over time, such as a prolonged activation of hypoxic pulmonary vasoconstriction (HPV), pulmonary hypertension (PH), right ventricular hypertrophy, and heart failure [3]. Chronic alveolar hypoxia is the cause of Group Three PH (WHO classification) and acts by inducing vasoconstriction as well as by stimulating the remodeling of small pulmonary arteries [4,5].

The recurrent night-time episodes of apnea/hypopnea observed in obstructive sleep apnea (OSA) patients with repeated cycles of hypoxia/reoxygenation, exemplify the existence of chronic intermittent hypoxia (CIH) in humans. Epidemiological studies indicate that chronic OSA plays a pathogenic role in cardiovascular disease [6]. CIH is associated with the development of systemic hypertension (HTN), left ventricular dysfunction, and stroke, as well as metabolic and neurological disorders in OSA patients [7,8,9,10]. Systemic hypertension and metabolic disorders have also been reported in CIH mouse and rat models [11,12,13] and it is well established that most of the pathology associated with OSA is a consequence of the intermittent activation of the carotid body [2,14,15].

Less consensus exists regarding the association between OSA and PH. Although the WHO categorizes sleep breathing disorders along with COPD as a cause of PH (see [16]), with alveolar hypoxia seen as being the cause of pulmonary arteriolar vasoconstriction, increased pulmonary arterial pressure (PAP), right ventricular afterload, and eventually cor pulmonale, this relationship has been questioned as inconsistent results were reported for the association between parameters of OSA severity and pulmonary hypertension. [17,18]. It has been estimated that most patients with OSA and no other major comorbidities have normal pulmonary artery pressures (PAP) and only about 10% of OSA patients exhibit a mild diurnal elevation of PAP. Therefore, CIH is seen as an exacerbating factor in patients with multifactorial PH, comorbidity with COPD, and obesity [17,18].

The remodeling of the pulmonary circulation has been investigated in animal models of OSA, in which other comorbidities are missing. In rodents, CIH raises PAP, produces right ventricular hypertrophy (RVH), and causes the muscularization of pulmonary arteries such as that caused by sustained hypoxia [10,13,19]. Unlike in the systemic circulation, no neurally mediated effect seems to play a significant role in the development of PH in response to CIH, and it seems that HPV, resulting from the brief and repetitive periods of intermittent hypoxia, would be sufficient for modifying the pulmonary vascular tone [18,19,20]. The molecular mechanism by which CIH would cause PH remains poorly characterized. Evidence supports a role for NADPH oxidase as an important source of superoxide in the vasculature, a well-recognized stimulus for vascular smooth muscle cell proliferation and vasoconstriction. The PH caused by CSH was abolished in mice lacking the gp91phox NADPH oxidase subunit [21] and CSH increased the expression of the Nox4 NADPH oxidase subunit in mice [22]. Superoxide reduces the NO bioavailability contributing to the endothelial dysfunction observed in PH [23] and there is evidence suggesting that recurrent cycles of hypoxia and re-oxygenation may cause PH via inflammatory pathways [17].

The aim of this work was to analyze the pulmonary and systemic cardiovascular responses in two rat models of PH, associated with CSH or CIH exposure, by comparing the hemodynamics, vascular contractility, and L-arginine-NO metabolism. We found that CIH rats develop systemic hypertension, also showing an increase in PAP but not right ventricular hypertrophy. In contrast, CSH exposure triggers PH and RVH, but not HTN. Pulmonary arteries evidenced more endothelial damage in animals exposed to CSH than to CIH, as they presented a reduced carbachol-mediated endothelial vasodilatation. Decreased plasma L-arginine/ADMA and L-arginine/SDMA ratios, which were found in CSH rats and a decreased pulmonary artery L-arginine/SDMA found in CIH rats, could contribute to the decreased pulmonary NO production and increased PAP. In summary, CSH and CIH seem to target different mechanisms for producing endothelial dysfunction and increased pulmonary and systemic blood pressure.

## 2. Materials and Methods

### 2.1. Animals and Anesthesia

Experiments were carried out in compliance with the international laws and policies (European Union Directive for Protection of Vertebrates Used for Experimental and Other Scientific Ends (2010/63/EU)) and approved by the University of Valladolid Institutional Committee for Animal Care and Use (Project Approval Ethical Code: 4,505,502).

Experiments were performed on male adult Wistar rats (3–4 months old) randomly distributed into three groups and housed under one of the following three experimental conditions: normoxic control rats (C group; *n* = 24), housed four per cage in the vivarium of the University of Valladolid, with free access to food and water, under controlled conditions of temperature and humidity. A second group of animals was identically maintained and fed but subjected to chronic intermittent hypoxia (CIH group; *n* = 16) as described previously [24]. Briefly, the protocol consisted of cycles of exposure for 40 s to 5% O_2_, then exposure to air for 80 s, repeating this cycle for 8 h each day (from 8:00 a.m. to 16:00 p.m., corresponding to the inactive period of animals, with SaO_2_ below 90% during 25% of the 8 h duration of the hypoxic exposure), for 14 days. A gas control delivery system regulated the flow of room air, N_2_, and O_2_ into the customized cages housing the rats. Otherwise, animals were exposed to room air. A third group of animals (CSH group; *n* = 16) consisted of rats, housed four per cage, introduced into a glass chamber (120 × 50 × 25 cm), where they were subjected to sustained hypoxia. The chamber was connected to a gas mixer adjusted to 11–12% O_2_ (concentration checked with external oximeter) with a constant flow of 3 L/min, with an outflow and soda lime (changed regularly when it reached its carbon dioxide absorbing capacity) to prevent CO_2_ and water vapor accumulation. The chamber was opened every 4 days for cleaning and feeding purposes, a procedure requiring about 30 min.

At the end of experiments, animals were euthanized by the administration of a lethal dose of sodium pentobarbital.

### 2.2. In Vivo Systemic and Pulmonary Arterial Pressure Measurements

Sodium pentobarbital anesthetized rats were tracheotomized and pump-ventilated (CL Palmer, London, UK) with room air (60 cycles/min and a positive expiratory pressure of 2 cm H_2_O). Systemic (SAP) and pulmonary arterial pressures (PAP) were continuously monitored with catheters inserted in the common carotid artery and pulmonary artery (PA), respectively, as previously described [25]. To reach the PA, a minimal thoracotomy was performed, a catheter was inserted into the right ventricle and, under oscilloscopic control, was driven to the PA. As with the SAP measurements, the catheter was connected to a pressure transducer (Transpac IV; ICU Medical, Inc., San Clemente, CA 92673, USA), with signals stored for subsequent analysis (heart rate, systolic and diastolic pressure, pulse pressure) using a BIOPAC system MP150. To record the effect of hypoxia on PAP, the inlet of the respirator was connected to a balloon filled with a mixture of 10%O_2_/90%N_2._ Animals in the CSH group were kept in hypoxia and then switched to normoxia immediately before these measurements were made.

### 2.3. Measurement of Right Ventricle (RV) and Left Ventricle (LV) Weights

Immediately after death, the heart and lungs were dissected out of the chest cavity. The cardiac atria were removed at the plane of the atrio-ventricular valves. The RV free wall was then carefully dissected from LV and septum (S) The RV and LV plus S were separately weighed, and the RV/(LV + S), the Fulton index, which is widely used as an indicator of RVH [13], was calculated in order to determine if this had occurred.

### 2.4. Hematocrit Measurement

Blood was collected by a carotid artery puncture immediately before death into a heparinized syringe; 100 µL of heparinized blood was placed in capillary tubes and hematocrit was determined using the standard technique.

### 2.5. Intrapulmonary Arteries Mounting and Measurement of Tension Development

The heart and lungs were excised and placed in cold physiological salt solution (PSS), which contained the following (mM): 118 NaCl, 24 NaHCO_3_, 1 MgSO_4_, 0.435 NaH_2_PO4, 5.5 glucose, 1.8 CaCl_2_, and 4 KCl. Rings of intrapulmonary arteries (IPA) (inner diameter 0.5–1.0 mm) were dissected free of adventitia and parenchyma under a dissection microscope, mounted on a conventional small vessel wire myograph, and stretched to give a basal tension of 5–6 mN (equivalent to an internal pressure of ~15 mmHg). They were then equilibrated with three brief exposures (3 min) to PSS containing 80 mM KCl (KPSS; isotonic replacement of NaCl by KCl). All experiments were conducted at 37 °C, with preparations gassed with 5%CO_2_/20%O_2_/balance N_2_. For the phenylephrine (PE) dose response experiments, cumulative concentrations of PE (from 0.01 to 3 µM) were added to the bath and for analysis, the resulting contraction was represented as a percent of the contraction measured after 3 min of exposure to KPSS. For the carbachol dose response relaxation, we first achieved a non-maximal stable PE contraction (60–70% maximal PE contraction). Once IPA reached this stable contraction, cumulative dose responses of carbachol (from 3 nM to 3 µM) were added to the tissue. L-NAME, the effect of which is irreversible, was added at a 100 µM concentration on the baseline at the end of the experiment and caused a contraction that was also quantified as the percent of that recorded at a 3-minute exposure to KPSS.

### 2.6. Preparation of Protein Extracts and Immunoblotting

Whole pulmonary arterial trees from the big pulmonary lobe were dissected free of adventitia and parenchyma, weighed and homogenized using a Dounce glass/glass homogenizer at 4 °C in RIPA buffer (10 mM Tris-HCl, pH 7.5, 300 mM NaCl, 3 mM MgCl_2_, 0.5% NP-40, 0.1% SDS, 0.5% sodium deoxycholate, 0.5 mM PMSF, 10 mM NaF, 10 μg/mL leupeptin, 10 μg/mL aprotinin, 2 mM orthovanadate), incubated at 4 °C for 1 h with gentle agitation, centrifuged at 15,000× *g* for 20 min, and the supernatant was stored at −80 °C until use. Samples (~20 µg protein/lane) were denatured by boiling in SDS sample buffer and loaded onto 10% polyacrylamide gels for SDS-PAGE. Gels were run at 50 mA and proteins transferred onto PVDF membranes (Amersham) at 100 mA for 1 h at 4 °C. Blots were blocked with 5% skimmed milk in Tris buffered saline with 0.1% Tween-20 (TTBS) for 1 h at room temperature, probed overnight at 4 °C with specific monoclonal antibodies for eNOS (1:500; BD, Biosciences, Madrid, Spain) and β-actin (1:2000; Sigma-Aldrich, Dorset, UK), and washed and probed with HRP-conjugated goat anti-mouse IgG (1:2000; BD, Biosciences, Madrid, Spain) for 1 h at room temperature. Immunoreactivity was visualized with enhanced chemiluminescence reagents (Amersham), and the signals were quantified by ImageJ analysis using optical density measurements. The data are presented as percent changes in immunoreactivity in hypoxic samples as compared to the immunoreactivity measured in normoxic samples (100%) as the eNOS area/β-actin area ratio in the same experiment and on the same blot.

### 2.7. Determination of Plasma Nitrites and Nitrates

Plasma was obtained by aortic puncture and stored at −80 °C. A method based on the Griess reaction was used to measure nitrites and nitrates as described in detail [26]. Briefly, plasma samples were incubated with nitrate reductase (Sigma-Aldrich, Dorset UK) for 2 h at room temperature; then, Griess reagent (Sigma-Aldrich, Dorset, UK) was added to the sample and the absorbance was read at 540 nm with a microplate reader 10 min later and interpolated to a standard curve with different concentrations of sodium nitrate (0–60 µM).

### 2.8. Measurement of Plasma Catecholamine Levels and Adrenal Medulla Catecholamine Content by HPLC-ECD

Plasma and isolated adrenal medulla were collected in EDTA precoated tubes and Eppendorf tubes, respectively. For catecholamine quantification, a two-step chromatographic method was applied. In the first step 400-microliter plasma samples were purified and catecholamines were extracted using 30-milligram OASIS HLB Wat cartridges (Waters, Waltham, MA, USA) and eluted in 500 μL of mobile phase. Then, a 100-microliter sample was directly injected into an HPLC system composed of a Waters 600 controller pump, a Waters C-18 (particle size 4 μm) column, a Waters 717 plus autosampler, and a Bioanalytical Systems LC-4A electrochemical detector (set at a holding potential of 0.65 mV and a sensitivity of 1 nA). The mobile phase consisted of a solution of 25 mM Na_2_HPO_4_, 0.65 mM 1-octane sodium sulfonate acid, 0.1 mM EDTA, pH 3.46, and 6% MeOH running at a flux of 1.0 mL/min. The signal from the detector was fed to an analog to digital converter controlled by Peak Sample Chromatography System Software (Buck Scientific, East Norwalk, CT, USA). Identification and quantification of catecholamines were conducted against external standards of norepinephrine (NE), epinephrine (E), and dopamine (DA) previously injected in the HPLC-ECD with known concentrations. Quantification was made with Peak Sample Data Chromatography System software (Buck Scientific, East Norwalk, CT, USA). 

For quantification of endogenous catecholamine content in adrenal medulla, frozen tissues were homogenized in 0.6 N perchloric acid (PCA) containing 0.1 mM EDTA and centrifuged at 12,000× *g* for 10 min. Supernatants were directly injected into the HPLC-ECD at the same conditions as the plasma. The values of amine concentrations were normalized to the tissue weight.

### 2.9. Measurements of L-Arginine and Metabolites

For the simultaneous analysis of the several endogenous substances involved in the NO-generating pathway, the plasma levels and pulmonary artery content of L-arginine, asymmetrical dimethyl-L-arginine (ADMA), and symmetrical dimethyl-L-arginine (SDMA) were measured by a two-step HPLC-FD with fluorescence detection. Plasma samples from the three animal groups were prepared adding 1 mM stock solution of the internal standard monomethyl-arginine (MMA) prepared in 10 mM HCl and using a pre-conditioning solid extraction cartridge (Oasis MCX cation-exchange SPE columns (30 mg, 1 cc) supplied by Waters as described [27].

Briefly, a 0.2-milliliter sample was mixed with 0.1 mL of internal standard (MMA) and 0.7 mL of PBS. The Oasis MCX SPE columns were preconditioned with 1 mL of ammonia/water/methanol (10/40/50) and 1 mL of water. After sample addition, columns were consecutively washed with 1 mL of 100 mM HCl and 1 mL of methanol. Analytes were eluted with 1 mL of ammonia/water/methanol (10/40/50). All washing and elution steps were performed by vacuum suction. Column eluates were evaporated under a vacuum lyophilizer and frozen at −20 °C until injection in the HPLC-FD. Before injection, the amino acids were derivatized with 0.1 mL of *o*-phthaldialdehyde (OPA) reagent containing 3-mercaptopropionic acid added to the residue. The derivatized amino acids were separated by isocratic reversed-phase chromatography performed on a C18 column (Strata-XL-C 100 µm, 30 mg/mL Phenomenex) using a mobile phase consisting of potassium phosphate buffer (50 mmol/L, pH 6.5), containing 8.7% acetonitrile at a flow rate of 0.3 mL/min. Fluorescence detection was performed at excitation and emission wavelengths of 340 and 455 nm, respectively.

Frozen rat tissue samples from isolated pulmonary arteries were weighed before analysis and, per gram tissue, 1 mL of 0.6 N perchloric acid was added. The samples were homogenized on ice using a glass-to-glass homogenizer, and subsequently centrifuged at 2000× *g,* 5 min at 4 °C. A 0.2-milliliter aliquot of the resulting supernatant was used for further analysis of methylated arginine as described for plasma samples, except for 10 µM L-homoarginine added as an internal standard. Peaks were quantified based on peak area.

### 2.10. Data Presentation and Statistical Analysis

Data were evaluated using GraphPad Prism Software, version 6 (GraphPad Software, La Jolla, CA, USA) and presented as mean values ± SEM. The significance of the differences between the mean values was calculated using the unpaired *t*-test, a one-way ANOVA with Tukey’s multiple comparison test, Dunnett’s multiple comparison test, and Sidak’s multiple comparison test and two-way ANOVA with Sidak’s multiple comparison test depending on the number of groups compared. Differences were considered statistically significant at a *p*-value of <0.05.

## 3. Results

### 3.1. Physiological Parameters: Body Weight, Hematocrit, and Hematological Parameters

To compare the effect of sustained vs. intermittent hypoxia on the physiological responses to chronic hypoxia, 3-month-old male rats were exposed to 10% O_2_ continually or 5–21% O_2_ (8 h/day) for 2 weeks. The rats were weighed, and blood was obtained for hematocrit and other physiological parameters at the end of hypoxic exposure. While the control animals demonstrated a 14.7% weight gain during the period of the study, the weight of CIH animals was almost unchanged (0.3% gain) and the CSH animals underwent a −9.9% loss of body weight (*p* < 0.001 for both hypoxic groups vs. the controls). Thus, in effect, both types of hypoxia led to a loss of weight relative to the controls. There was also a significant difference with respect to the development of polycythemia: the rats exposed to CSH but not CIH developed significant polycythemia and increased blood hemoglobin levels compared with normoxic rats. However, both treatments increased hematocrit, the increase in CIH (44.6 ± 0.5%; *p* < 0.05) being lower than in CSH rats (54.5 ± 1.0%; *p* < 0.001), compared with the control values (42.8 ± 0.6%), as shown in Table 1.

### 3.2. Systemic and Pulmonary Hemodynamics

**Figure 1:**
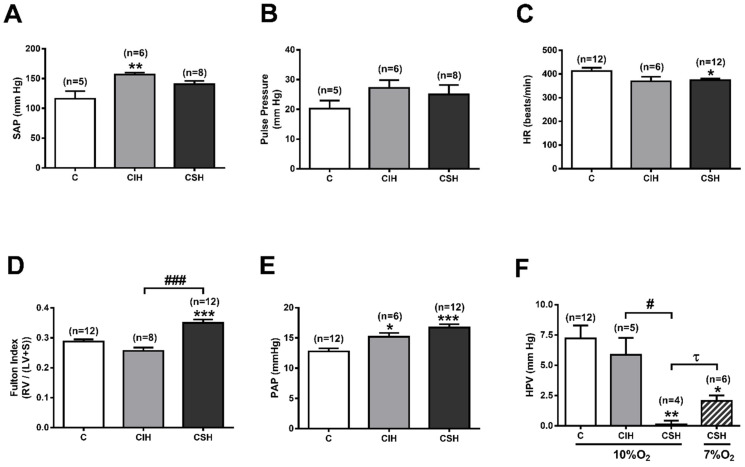
Hemodynamic measurements. (**A**) Mean systemic arterial pressure (SAP; mmHg) in C (control), CIH, and CSH animals. Data are expressed as mean ± SEM; ** *p* < 0.01, vs. C animals (one-way ANOVA with Tukey’s multiple comparison test). (**B**) Pulse pressure (systolic minus diastolic pressure; mmHg) in C, CIH, and CSH animals. (**C**) Heart rate, as beats per minute, in C, CIH, and CSH animals; * *p* < 0.05 vs. C animals (one-way ANOVA with Tukey’s multiple comparison test). (**D**) Fulton index (Right ventricle/ (Left ventricle + Septum)) in C, CIH, and CSH animals. Data are expressed as mean ± SEM; *** *p* < 0.001 vs. C animals (one-way ANOVA with Tukey’s multiple comparison test); ### *p* < 0.001 CIH vs. CSH animals (unpaired *t*-test). (**E**) Mean pulmonary arterial pressure (PAP; mmHg) in C, CIH, and CSH animals. Data are expressed as mean ± SEM; * *p* < 0.05, *** *p* < 0.001 vs. C animals (one-way ANOVA with Tukey’s multiple comparison test); (**F**) Hypoxic pulmonary vasoconstriction response (HPV) to 10% O_2_ in C, CIH, and CSH animals, and to 7% O_2_ in CSH animals. Data expressed as mean ± SEM; * *p* < 0.05, ** *p* < 0.01 vs. C animals (one-way ANOVA with Tukey’s multiple comparison test); # *p* < 0.05 CIH vs. CSH animals (unpaired *t*-test); ^τ^ *p* < 0.05 CSH 10% O_2_ vs. CSH 7% O_2_ (unpaired *t*-test).

To assess the impact of CIH and CSH exposure on systemic and pulmonary hemodynamics, direct measurements of arterial pressure on the carotid and pulmonary artery were obtained by catheterization in rats ventilated with 21% O_2,_ except during the measurements shown in Figure 1F. When compared with the control group, CIH exposure increased the mean systemic arterial blood pressure (SAP) from 116 ± 13 to 157 ± 3 mmHg (*p* < 0.01; Figure 1A). A non-significant increase was observed in the CSH group (141 ± 6 mmHg; *p* > 0.05). The pulse pressure did not change significantly in any of the experimental groups, although it tended to increase slightly in both hypoxic groups (23–34%; Figure 1B). A significantly decreased heart rate was seen in CSH rats but not CIH rats compared to the control group (413 ± 14 bpm; Figure 1C).

To determine the impact of blood pressure alterations on ventricular cardiac mass, RV and LV plus S weights were measured (Figure 1D). The rats exposed to CSH had an increased RV mass, expressed as the Fulton index (0.35 ± 0.01), compared to normoxic rats (0.29 ± 0.01; *p* < 0.05), whereas there was no significant change in the CIH group. These results indicate right ventricular hypertrophy only in the CSH group and suggest left ventricular hypertrophy in the CIH group. When calculated as LV + S/whole heart weight (data not shown), the ratio increased in the animals exposed to CIH (0.80 ± 0.01 vs. 0.78 ± 0.00; *p* < 0.05) and did not change significantly in the CSH group. Therefore, the development of both polycythemia and RV hypertrophy, two well-documented responses to sustained chronic hypoxia, did not appear when intermittent chronic hypoxia was applied. To determine the possible relationship between the type of hypoxia, RV hypertrophy, and pulmonary hypertension, PA pressure was measured directly by catheterization (Figure 1E) and was found to be significantly increased by both types of hypoxia. Mean PAP was 12.8 ± 0.5 mmHg in controls, 15.2 ± 0.6 mmHg in CIH rats (*p* < 0.05 vs. controls), and 16.7 ± 0.6 mmHg in CSH animals (*p* < 0.001 vs. controls).

Finally, to assess if the HPV response was maintained after hypoxic treatment, PAP was measured while rats were ventilated with 10% O_2_ for 3 min by mechanical ventilation (Figure 1F). PAP was augmented in controls by 7.2 ± 1.1 mmHg and 5.9 ± 1.4 mmHg in CIH rats. In CSH animals, the same hypoxic challenge produced no change in PAP (0.1 ± 0.3 mmHg). In a second set of experiments, we tested the possibility that the reduced response to hypoxia observed in the CSH rats could be due to the use of a hypoxic stimulus very similar to that used in the chronic treatment (11% O_2)_. Thus, we additionally tested the HPV in CSH rats with a 7% O_2_ gas mixture, finding that the PAP increased by 2.1 ± 0.4 mmHg (*p* < 0.05 vs. 10% O_2_).

### 3.3. Pulmonary Vascular Contractility

**Figure 2:**
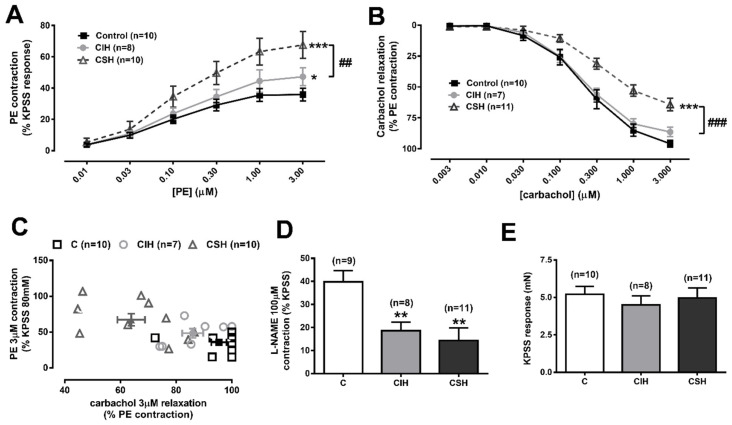
Intrapulmonary artery (IPA) wire myography. (**A**) Phenylephrine (PE) dose response (0.01–3 µM) as % of the KPSS response, in C, CIH, and CSH arteries; Data are expressed as mean ± SEM; * *p* < 0.05, *** *p* < 0.001 vs. control curve (two-way ANOVA with Sidak multi comparison test); ## *p* < 0.01 CIH vs. CSH. (**B**) Dose response relaxation to carbachol (0.003–3 µM) as % of a stable submaximal PE contraction in C, CIH, and CSH arteries; Data are expressed as mean ± SEM; *** *p* < 0.001 vs. control curve (two-way ANOVA with Sidak multi comparison test); ### *p* < 0.001 CIH vs. CSH. (**C**) Interpolation of the maximal contraction to PE (Y-axis) with the maximal relaxation to carbachol (X-axis) for each individual artery, with the average values for each animal group. (**D**) 100µM L-NAME responses, expressed as % of the KPSS response in C, CIH, and CSH; Data are expressed as mean ± SEM; ** *p* < 0.01 vs. C rats (one-way ANOVA with Tukey multi comparison test). (**E**) Response of IPA to 3 min of PSS with 80 mM K^+^ (KPSS), expressed in mN, and in C, CIH, and CSH arteries. Data are expressed as mean ± SEM.

The difference in HPV between CIH and CSH could reflect the altered vasomotor and/or vasoproliferative responses to chronic hypoxia. Figure 2A shows the vascular contractile responses to cumulative doses of phenylephrine in the small PA. Contraction induced by PE was greater in the PA of animals exposed to hypoxia, both in the CIH (*p* < 0.05), or more markedly in the CSH group (*p* < 0.001 vs. control; *p* < 0.05 vs. CIH) than in the control group. Figure 2B shows the changes in tension of the PA caused by cumulative doses of carbachol (3 nM–3 µM), after pre-contraction with a submaximal stable PE concentration, which was matched among individual arteries (60–70% maximal PE contraction). This protocol was used as a test for endothelial functionality, as carbachol releases NO from the endothelial layer of the artery. No differences in the endothelium-dependent responses were observed in CIH arteries compared to the controls. Interestingly, in CSH arteries, the endothelium dependent vasorelaxation was significantly blunted (*p* < 0.001) with respect to the controls, indicating the existence of endothelial dysfunction in the PA. Figure 2C shows the relationship between the maximal contraction to PE (data from Figure 2A, on the *Y*-axis) and the maximal relaxation to carbachol (data from Figure 2B, on the *X*-axis) for each individual artery (*n* = 27 xy pairs), and the mean values with standard errors for each animal group. The resulting correlation analysis of both variables yielded a Pearson correlation coefficient *r* = −0.5494 that was statistically different from zero (*p* = 0.0030), indicating that there is a moderate inverse relationship between the contraction to one stimulus and the relaxation to the other. When we applied the eNOS inhibitor L-NAME (100 µM), the resultant contraction was significantly lower in both hypoxic groups ((Figure 2D), with a reduction of 53% in CIH and 64% in CSH; *p* < 0.01 for both groups). The contraction induced by KPSS was similar in the PA of the animals exposed to CSH or CIH, compared to the controls (Figure 2E).

Taken together, these data suggest a diminished availability of endothelial NO in the arteries after hypoxic exposure (CSH and CIH), possibly resulting in a higher vascular tone than in control arteries that could be responsible for the higher PAP (Figure 1E).

### 3.4. Plasma and Adrenal Medulla Catecholamine Levels

**Figure 3:**
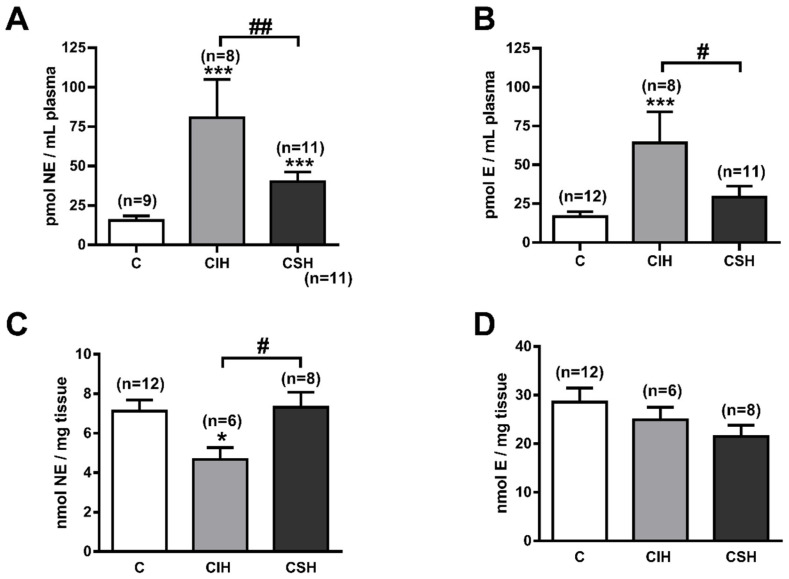
Plasma and adrenal medulla catecholamine content. (**A**) Endogenous content of plasma norepinephrine (NE; pmol/mL) from C, CIH, and CSH rats. Data are expressed as mean ± SEM; *** *p* < 0.001 vs. C. One-way ANOVA with Tukey’s multiple comparisons test: ## *p* < 0.01 CIH vs. CSH, One-way ANOVA with Sidak’s multiple comparisons test. (**B**) Endogenous content of plasma epinephrine (E; pmol/mL) from C, CIH, and CSH rats. Data are expressed as mean ± SEM; *** *p* < 0.001 vs. C. One-way ANOVA with Tukey’s multiple comparisons test: # *p* < 0.05 CIH vs. CSH One-way ANOVA. (**C**) Endogenous content of adrenal medulla norepinephrine (NE; nmol/mg tissue) from C, CIH, and CSH rats. Data are expressed as mean ± SEM; * *p* < 0.05 vs. C. One-way ANOVA with Tukey’s multiple comparisons test: # *p* < 0.05 CIH vs. CSH One-way ANOVA. (**D**) Endogenous content of adrenal medulla epinephrine (E; nmol/mg tissue) from C, CIH, and CSH rats. Data are expressed as mean ± SEM.

To establish a possible correlation between sympathetic activity and PAP, we have analyzed circulating catecholamines in the three groups of rats. As sympathetic nerve endings are the main source (80%) of plasma norepinephrine (NE), and epinephrine (E) comes entirely from the adrenal medulla, the levels of NE and E represent a suitable index of the generalized sympathetic tone of mammalian organisms [28]. The plasma NE levels increased significantly in the two experimental groups but with a greater increase of 80.7 ± 24.3 pmol/mL in the CIH animals vs. 40.0 ± 6.2 pmol/mL in the CSH group (*p* < 0.05) compared to 15.4 ± 2.9 pmol/mL in the control group (both, *p* < 0.001; Figure 3A). The plasma E levels showed a very similar pattern to those of NE (Figure 3B), also showing significant differences between CIH and CSH (*p* < 0.05). No significant changes occurred in the NE and E content in the adrenal medulla from experimental groups except for a decreased NE content in the CIH (4.7 ± 0.6 nmol/mg tissue) vs. C (7.1 ± 0.6 nmol/mg tissue) and CSH (7.3 ± 0.7 nmol/mg tissue) groups (*p* < 0.05; Figure 3C), although there was a trend for adrenal E to decrease in the two experimental groups (Figure 3D).

### 3.5. Nitrites and Nitrates Plasma Levels and Expression of Vascular eNOS

**Figure 4:**
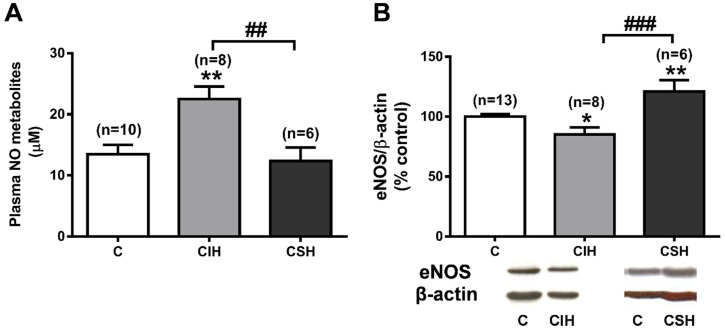
NO metabolites plasma levels and eNOS expression (WB) in pulmonary arteries. (**A**) Plasma levels of nitrite/nitrates, expressed in µM, in C, CIH, and CSH. Data are means ± SEM from *n* = 6–12 rats. ** *p* < 0.01 vs. C animals, one-way ANOVA with Tukey multi comparison test; ## *p* < 0.01 CIH vs. CSH unpaired t-test. (**B**) Representative Western blot obtained with eNOS antibody (dilution 1:500) showing a single band of expected molecular weight (130 kDa) in PA from C, CIH, and CSH. Optical densities of eNOS were quantified, normalized relative to β-actin signal (eNOS/β-actin), averaged for all the replicated gels, and expressed as % of the control arteries (set as 100%), in CIH and CSH pulmonary arteries. Values are mean ± SEM (*n* = 6–8 arteries); * *p* < 0.05 vs. C; ### *p* < 0.001 CIH vs. CSH; one-way ANOVA with Tukey multi comparison test.

To assess the availability of NO after hypoxic treatment, the plasma levels of nitrites, and the nitrates were measured as an index of vascular NO production [29]. NO metabolites were examined by converting nitrate into nitrite by a nitrate reductase catalyzed reaction and then assessing nitrite using spectrophotometry after the addition of Griess reagent. As shown in Figure 4A, CIH exposure increased NOx levels by 66.7% (22.5 ± 2.1 vs. 13.5 ± 1.6 µM), whereas CSH exposure did not change them when compared to control animals (12.4 ± 2.2 vs. 13.5 ± 1.6 µM). However, the effects of CSH and CIH on plasma NOx were not explained by a change in the eNOS protein expression in the PA (Figure 4B), as CIH decreased the expression of eNOS by 15% (*p* < 0.05) vs. the control group (taken as 100% eNOS/β-actin), whereas CSH exposure significantly increased the eNOS protein expression (121 ± 10%; *p* < 0.01).

### 3.6. Plasma and Pulmonary Artery L-Arginine and Methylarginines

**Figure 5:**
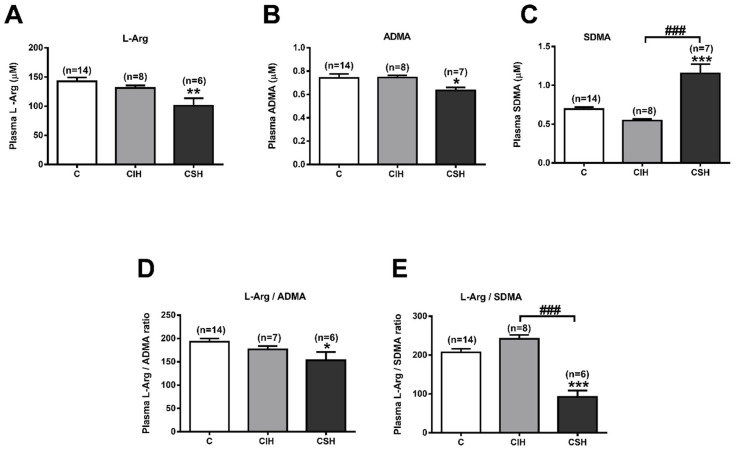
L-Arginine and arginine-methylated metabolites in plasma. (**A**) Plasma levels of L-Arginine (in µM). (**B**) Plasma levels of ADMA (in µM). (**C**) Plasma levels of SDMA (in µM) measured by HPLC-FD. (**D**). Ratio L-Arginine/ADMA. (**E**). Ratio L-Arginine/SDMA in C, CIH, and CSH rats. Data are expressed as mean ± SEM (*n* = 8–14). * *p* < 0.05, ** *p* < 0.01, *** *p* < 0.001 vs. C one-way ANOVA with Tukey multi comparison test; ### *p* < 0.001 CIH vs. CSH, One Way ANOVA.

Endogenous inhibitors of NOS could affect the synthesis of NO. ADMA is the main endogenous eNOS inhibitor, whereas the closely related compound SDMA does not inhibit NOS, but competes with arginine for cellular uptake, thereby limiting the substrate availability for NOS. We have simultaneously analyzed the plasma levels of L-arginine, ADMA, and SMDA from CIH and CSH rats, and compared them to the control values. Figure 5A shows that CSH exposure decreases plasma L-arginine from 142 ± 78 µM in control rats to 101 ± 13 µM (*p* < 0.01), whereas CIH did not modify it (131 ± 4; *p* > 0.05). There were significant differences in the plasma levels of ADMA from the CIH or CSH groups vs. the control group (*p* < 0.05; Figure 5B). Figure 5C shows increased SDMA plasma levels in CSH (1.2 ± 0.1 vs. 0.7 ± 0.0 µM in control group; *p* < 0.001) and no change in the CIH group. The L-arginine/ADMA and L-arginine/SDMA ratios after CSH exposure decreased by 20% (*p* < 0.05) and 55% (*p* < 0.001), respectively, compared to the control ratio (Figure 5D,E). Both ratios remained unchanged after CIH exposure.

**Figure 6:**
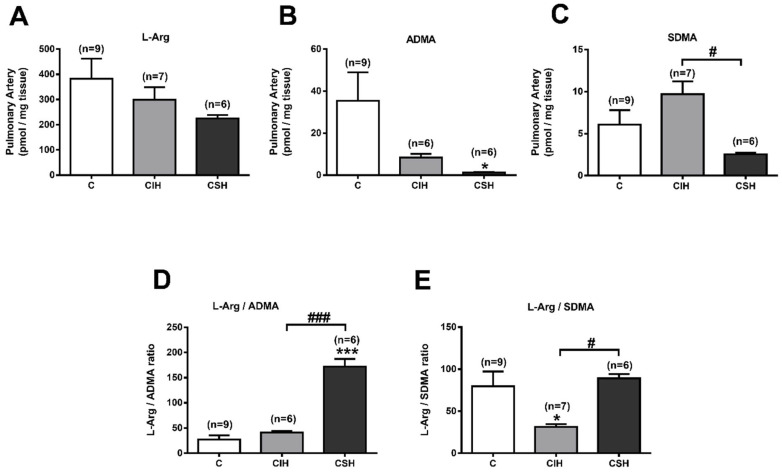
L-Arginine and arginine-methylated metabolites in pulmonary arteries (PA). (**A**) PA levels of L-Arginine (pmol/mg tissue). (**B**) PA levels of ADMA (pmol/mg tissue). (**C**) PA levels of SDMA (pmol/mg protein) measured by HPLC-FD. (**D**) Ratio L-Arginine/ADMA. (**E**) Ratio L-Arginine/SDMA, in C, CIH, and CSH animals. Data are expressed as mean ± SEM (*n* = 6–9 rats). * *p* < 0.05, *** *p* < 0.001 vs. C one-way ANOVA with Dunnet post-test; # *p* < 0.05, ### *p* < 0.001 vs. CIH unpaired t-test.

Hypoxia evoked a similar pattern for the concentration of L-arginine in pulmonary arteries as in the plasma: it tended to decrease in both the CIH and CSH groups, although its effects were not significant in either group (Figure 6A; *p* > 0.05). The concentration of ADMA in both experimental groups (Figure 6B) decreased from 35.4 ± 13.5 pmol/mg PA in the control group to 8.4 ± 1.7 and 1.3 ± 0.1 pmol/mg PA in CIH and CSH, respectively. Conversely, the SDMA levels (Figure 6C) tend to increase in the CIH group (9.7 ± 1.5 pmol/mg PA), while they tend to diminish in the CSH group (2.6 ± 0.2 pmol/mg PA) vs. the control group (6.1 ± 1.7 pmol/mg PA). L-Arg/ADMA ratio (Figure 6D) increases in the CSH group (171.8 ± 15.4 vs. 26.9 ± 8.7 in C, *p* < 0.001) while the L-Arg/SDMA ratio (Figure 6E) decreases in the CIH group (31.5 ± 3.1 vs. 79.7 ± 17.6 in C group, *p* < 0.05).

## 4. Discussion

**Figure 7:**
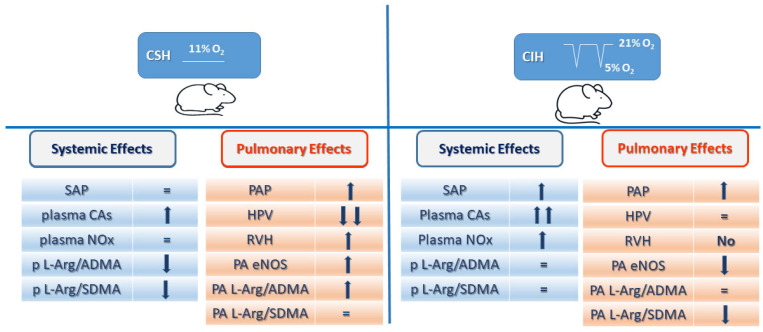
Schematic figure representing the main findings on systemic and pulmonary effects in rats exposed to CSH (**left**) and CIH (**right**) vs. Control rats. SAP: systemic arterial pressure; CA: catecholamine; NOx: Nitrites; ADMA: asymmetrical dimethyl-L-arginine; SDMA: symmetrical dimethyl-L-arginine; PAP: pulmonary arterial pressure; HPV: hypoxic pulmonary vasoconstriction; RVH: Right ventricular hypertrophy (Fulton index); PA: pulmonary arteries; p = plasma.

We hypothesized that chronic hypoxia, whether sustained or intermittent, would produce similar pulmonary vascular effects leading to endothelial dysfunction and PH and RV hypertrophy. To test the hypothesis, we compared the effects of CIH on pulmonary hemodynamics and function with those of CSH using aged-matched rats subjected to the same environmental conditions except for the type of hypoxic exposure. Figure 7 summarizes our results, and the potential molecular pathways that may contribute to CSH and CIH, as described below.

The clinical consequences of chronic hypoxia with sustained alveolar hypoxia, which occurs in COPD and other respiratory disorders, include maladaptive responses such as polycythemia, weight loss, PH, right ventricular hypertrophy (RVH), and an increased amount of smooth muscle cells (SMCs) in the distal pulmonary arterial branches. Animal models of CSH manifest similar physiological responses [30], many of which we corroborated in this study. However, the most common form of chronic hypoxia in humans is intermittent hypoxia due to obstructive sleep apnea (OSA), which has also been reported to cause polycythemia [31] and to contribute to the development of PH and RVH through pulmonary vasoconstriction secondary to hypoxia, although other studies indicate that intermittent hypoxia is not sufficient to cause sustained PH [32,33].

Our study corroborates previous observations that in animal models, i.e., in the absence of other pathologies, both CSH and CIH produce an increase in basal PAP, which is evident even when this is measured under normoxic conditions. Fagan [19] found that CSH caused a greater increase in PAP than CIH did, and Snow et al. [34] demonstrated that CSH but not CIH increased pulmonary vascular resistance in isolated lung. Right ventricular hypertrophy, which develops as a result of an elevated PAP, has also been seen to be increased to a greater extent in studies in which CSH was compared directly to CIH [19]. These findings suggest that whereas experimental CSH unequivocally increases PAP, the effect of CIH is smaller and may not always occur. In accordance with this concept, we saw that the effect of CIH on PAP appeared to be smaller, although this was not statistically significant, and that CSH increased the Fulton index, a measure of RVH, whereas CIH did not. This may have been due to an increase in the weight of the LV, which typically occurs in CIH [14] due to an increase in the systemic MABP, and which we also observed.

### 4.1. Effects of CSH and CIH on PA Responsiveness to Phenylephrine and Carbachol

The increase in PAP observed in animal models of CSH is due to PA remodeling, as well as pro-contractile changes in pulmonary vascular smooth muscle cells such as membrane depolarization, the upregulation of non-voltage gated Ca^2+^ entry pathways, and the enhancement of rho kinase- dependent Ca^2+^ sensitization. The mechanisms inducing these changes include the activation of HIF_1a_, HIF_2a_, and mTORC [35], as well as increases in ROS production by NADPH oxidase and mitochondria, possibly acting in concert with a decrease in the [superoxide]/[H_2_O_2_] ratio in the PA [36]. The increase in the baseline PAP caused by CSH is also generally seen as being dependent on an attenuation of NO-induced vasodilation together with an increased release of, and responsiveness to, ET-1 [37,38], although there have been reports that vasorelaxation via endothelial NO release is increased in animals subjected to CSH [34,39]. CSH also upregulates the activity of protein kinase G1a in PASMCs, an effect that depends on the ROS derived from NOX4. This represents a compensatory pathway that ameliorates both vasoconstriction and pulmonary vascular remodeling and fibrosis [40]. 

Our study utilized chronic periodic rapid oscillations of the pO_2_ to mimic OSA. Relatively few studies employing this type of protocol have examined its effects on the pulmonary circulation; therefore, although it is generally thought to cause a rise in PAP, the mechanisms responsible for this effect are not well understood. However, there is evidence that, similar to CSH, CIH causes pulmonary vascular remodeling [23,34,41]. Wang et al. [42] reported that CIH increased the expression of ET-1 and ET_A_ receptors in PASMC, whereas that of (vasodilating) ET_B_ receptors in pulmonary artery endothelial cells were diminished; they also observed increases in the efficacy and potency of ET-1 as a PA vasoconstrictor. While ET-1 causes Ca^2+^ sensitization in the PA by stimulating RhoA/ROK following CSH [43], the PA from rats exposed to CIH exhibit an augmented vasoconstricting ET-1-dependent Ca^2+^ sensitization due to PKCβ activation [44]. Snow et al. [34] also found that U46619-induced PA constriction was increased by CIH, although this effect was small, and was not observed when UTP was used as the vasoconstrictor [45]. CIH also causes an increased expression of NADPH oxidase and enhanced superoxide production by lung tissue [23], and an isolated PA [45].

We observed that the response of an isolated PA to PE, which stimulates arterial contraction largely via α_1_ receptors, was enhanced in both the CSH- and CIH-treated animals compared to the controls, although this effect was smaller in the CIH group. CSH has been shown to potentiate contractions of the PA evoked by several vasoconstrictors, including ET-1, angiotensin 2 [37], and 5-HT [46], whereas CIH has been shown to increase the response of a PA to U46619 [34] and ET-1 [42]. As far as we are aware, this is the first report that the response to an α_1_ receptor agonist is also enhanced by both types of hypoxic protocol. The larger effect on the PE contraction induced in A PA by CSH compared to CIH echoes a similar observation [34] that CSH increased the PA constriction elicited by the TP receptor agonist U46619 more than CIH did.

Conversely, in our experiments, the high K^+^ contraction was not increased by either hypoxic treatment. The apparently selective effect of CSH on the response to PE fits with previous evidence that sustained hypoxia increases the PA contraction by upregulating non-voltage dependent Ca^2+^ entry pathways and rho kinase activity, and down-regulating K_V_ channel expression [36], since these effects would be predicted to enhance the responses to G_q/11_ coupled receptors but not to high K^+^, which causes contraction by opening voltage-gated Ca^2+^ channels. There have been few studies of the mechanisms by which CIH increases the response of a PA to vasoconstrictors, but Snow et al. [47] have recently provided evidence that CIH potentiates the ET-1 contraction in a PA via a pathway involving the activation of protein kinase Cβ and mitochondrial ROS production.

### 4.2. Effects of CSH and CIH on NO, Methylated Arginines, and Endothelium-Dependent Relaxation of PA

CSH is generally viewed as causing a decrease in endothelium-dependent, NO-mediated vasodilation of PA, which contributes to the increase in basal PAP and the responsiveness to the vasoconstricting stimuli it elicits [37,38], although the opposite effect has also been reported [39,48]. While some have found that CSH decreases the expression of eNOS in the PA [49], an enhancement of eNOS expression has been more commonly reported [48,50]. Giaid and Saleh [51] found that eNOS immunostaining was significantly reduced in the lungs of humans with PH, although whether the loss of eNOS occurs in all patients with PH is uncertain and was questioned by other investigators [52]. Regardless of how it affects eNOS expression in PA, there are thought to be multiple mechanisms by which CSH depresses NO-mediated vasodilation in these arteries. These include greater NO scavenging by superoxide, eNOS uncoupling, which may also be the result of the higher pulmonary superoxide levels caused by CSH, and also an HIF-2-dependent increase in the expression of PA endothelial arginase II, which reduces the cellular concentration of arginine available for NO synthesis [38]. There is less information and no consensus on how CIH affects endothelial function. Snow et al. [34] observed a substantial CIH-induced increase in endothelium-dependent PA dilation, whereas Norton et al. [45], using the same model of CIH, found no effect on the response to ionomycin or on eNOS expression in PA. However, the application of the superoxide scavenger Tiron revealed an enhanced response to ionomycin, leading the authors to propose that CIH was causing an underlying increase in the responsiveness of PASMC to NO and cyclic GMP, which was masked by NO scavenging by superoxide. Conversely, Wang et al. [42] found that endothelium-dependent PA relaxation was slightly but significantly inhibited in their model of CIH. We found that CSH greatly diminished the contraction of the PA evoked by the eNOS antagonist L-NAME (Figure 2D). This effect has been observed by others [53] and has been interpreted as indicating that CSH depresses basal NO release. As other groups have also reported [37], we found that CSH also strongly depressed the carbachol-induced endothelium-dependent vasorelaxation of the PA (Figure 2B).

Our results support the concept that CSH leads to PA endothelial dysfunction and demonstrate that this can occur even though eNOS expression is increased (Figure 4B). One potential explanation for the suppression of the NO-induced vasodilation of the PA is that the concentration of methylated arginines in PA endothelial cells might be increased. Asymmetric dimethylarginine (ADMA) and its stereoisomer, symmetric dimethylarginine (SDMA), are formed by the post-translational methylation of L-arginine residues in proteins by protein arginine methyltransferases (PMRT). ADMA acts as an endogenous competitive inhibitor of NOS [54,55,56], whereas SDMA does not inhibit eNOS, but inhibits NO production by competing with L-arginine for cellular uptake. Indeed, Yaman et al. [57] found that plasma (ADMA) rose from 11 to 18 nmol/L in rabbits subjected to 30 days of CSH, Millat et al. [58] reported that one week of CSH in rats increased the ADMA content in rat lung homogenates by two to three-fold, and Luneburg et al. [59] found a similar increase in the ADMA levels in lung homogenate following 30 days of CSH; this was accompanied by a fall in the L-arginine to ADMA ratio. These results suggest that ADMA could contribute to endothelial dysfunction in both PA and systemic arteries. In contrast, we observed that CSH caused a profound fall in the ADMA content of PA, suggesting that this form of methylated arginine was not responsible for the loss of endothelium-mediated vasodilation. One potential reason for the discrepancy between our results and those reported previously is that the ADMA content of lung homogenates would also include that present in the airways, which could be increased in lung disease [60]. We found that the SDMA content of the PA was not affected by CSH (Figure 6C). However, CSH caused a substantial rise in the plasma (SDMA) (Figure 5C), with a fall in the L-arginine/SDMA ratio (Figure 5E). This could have contributed to PA endothelial dysfunction and the rise in PAP, although the relative impact of plasma vs. cellular SDMA concentrations on NO synthesis is not known.

We found that CIH had no significant effect on carbachol-induced vasorelaxation (Figure 2B) but caused a suppression of the L-NAME-induced contraction, the extent of which was similar to that seen with CSH (Figure 2C). This would imply that the mechanisms governing the basal and stimulated release of NO by these arteries differ, at least in some respects, and that only the former are affected by CIH. CIH caused a small but significant decrease in the eNOS expression in PA, which may have contributed to this. 

The effect of the type of CIH we used on the methylated arginine levels in the lungs appears not to have been previously described, but Badran et al. [61] observed that CIH (60 cycles/h for 8 weeks) increased the plasma ADMA concentration by ~40%. Interestingly, although we did not see any change in the plasma ADMA concentration, there was a small decrease in the L-arginine to ADMA ratio. Moreover, although CIH had no significant effect on either the SDMA or ADMA contents of the PA, it caused a large increase in the plasma SDMA concentration and a fall in the plasma (arginine); therefore, that the L-arginine/ SDMA ratio fell by more than 50%. This may have contributed to the basal endothelial dysfunction of the PA and to the rise in PAP and the systemic BP, although this remains to be confirmed.

There is no consensus regarding the effect of CSH on the plasma levels of NO metabolites (nitrites and nitrates; NOx), which are typically measured as an index of overall vascular NO bioavailability [29]. While Fagan et al. [62] reported that 10 days of CSH in rats reduced the plasma nitrate concentration from 6 to 4.5 mM, Bagali et al. [63] found a fall in plasma NOx in rats subjected to 21 days of CSH and Reinero et al. [64] saw no significant change in plasma NOx after 14 days of CSH. In agreement with this study, we also found no changes in the level of plasma NOx.

Conversely, we observed that plasma NOx was higher in the CIH rats (Figure 4A). A similar effect was observed by Bertuglia [65] in guinea pigs after 21 days of CIH. Activation of the nitrate-nitrite-NO pathway is an alternative source of NO, which may be particularly effective in the conditions of hypoxia and acidosis hat occurs in skeletal muscle during exercise [66] and could be the source of the increase in circulating NO. Although this might be predicted to lower PAP, the decreased expression of the eNOS protein in the PA that we observed, as well as pulmonary-specific effects such as NO scavenging, could oppose any vasodilating effect of higher circulating NO on the PA in CIH.

As shown in Figure 2C, there was a significant inverse correlation between the amplitude of the PE-induced contraction and the carbachol-induced relaxation of the isolated PA when the results from all three groups of rats were included. This suggests that the decrement in endothelium-dependent vasodilation contributes to the increased responsiveness to PE, possibly because vasoconstricting agonists evoke a rise in endothelial [Ca^2+^]_i_, which induces an NO release [67], thereby causing a suppression of contraction that would be diminished by endothelial dysfunction. This could explain how the marked loss of the endothelium-dependent relaxation in the PA caused by CSH could promote the increased contraction to PE, but not necessarily affect the basal NO release, such that the response to L-NAME was similarly decreased in CSH- and CIH-treated rats.

### 4.3. Effects of CSH and CIH on HPV

HPV, an acute constriction of the PA to alveolar hypoxia that diverts blood to well-oxygenated lung segments, exerts a beneficial effect by optimizing the ventilation-perfusion matching and systemic oxygen delivery [68]. However, sustained HPV due to global and persistent alveolar hypoxia, such as in chronic lung disease or at altitude, contributes to the development of Group Three PH [69]. The effects of CSH and CIH on HPV elicited under standardized conditions have not previously been compared in a single study. We observed that HPV evoked in response to 10% O_2_ was abolished in the CSH animals and was small even at a more severe level of hypoxia (Figure 1F).

The effect of CSH we observed agrees with previous findings [70]. The reasons that it suppresses HPV remain obscure, although it has been proposed to be due to the decreased K^+^ channel expression [71]. Little is known about the effects of CIH on HPV. However, it was reported by Shirai et al. [72] that HPV evoked by 8% O_2_ was greatly diminished in rats subjected to CIH (20 cycles of 4% O_2_/air per hour, 8 h a day for 6 weeks). Nagai et al. [73] later provided evidence that this was due to CIH causing the migration into the lung of macrophages in which iNOS and the β_3_ receptor were upregulated, such that sympathetic activation due to the CIH activation of these receptors was leading to an increased iNOS-mediated NO production, which suppressed HPV. In contrast, we found that the amplitude of HPV to 10% O_2_ in the CIH treatment group was similar to that in the normoxic controls (Figure 1F). It is possible that these divergent findings could be due to differences in the CIH protocols used. There is evidence, however, that PAP rises during episodes of nocturnal O_2_ desaturation in those with OSA [74], suggesting that the preservation of HPV we observed resembles what is occurring in this human form of CIH.

## 5. Conclusions

It is noteworthy that our results differ in some ways from those reported previously, and it seems most likely that this is due to the methodological variability between different studies, for example, in the addition (or not) of CO_2_ to the hypoxic gas mixture [34], the overall duration of the hypoxic interventions, and the timing of the oscillations of pO_2_ imposed during CIH. In summary, however, the general picture that emerges from our results is that, whereas CSH and CIH cause a number of common effects such as increased hematocrit, weight loss, and an increase in PAP, compared to CIH, CSH seems to have more of an effect on the pulmonary circulation, whereas the effects of CIH are apparently more targeted on the systemic circulation (Figure 7). CSH causes right ventricular hypertrophy, the abolition of HPV, marked changes in PA vasoreactivity, and a fall in the ADMA content of PA, but has no or minor effects on SAP, plasma NOx, or plasma catecholamines. Conversely, CIH increases SAP and plasma catecholamines and NOx, but has no effect on HPV, the Fulton index, the ADMA content of PA, or the endothelium-dependent relaxation of these arteries, and only slightly enhances PE-induced contraction. The results suggest that the endothelial dysfunction evident in the PA with both hypoxia protocols are not due to an increase in the methylated arginines in these arteries, although an increase in plasma (SDMA) could contribute to the apparent loss of basal NO-dependent vasodilation and, therefore, the increase in PAP that results from CIH. 

## Figures and Tables

**Table 1:** Endpoint Physiological Variables. Weight changes and hematological parameters in rats exposed to normoxia (C), chronic intermittent hypoxia (CIH), and chronic sustained hypoxia (CSH).

	C	CIH	CSH
Body weight change (%) ^1^	14.7 ± 1.4 (*n* = 8)	0.3 ± 0.6 *** (*n* = 8)	–9.9 ± 0.5 *** (*n* = 14)
Hematocrit (%)	42.8 ± 0.6 (*n* = 24)	44.6 ± 0.5 * (*n* = 16)	54.5 ± 1.0 *** (*n* = 12)
Erythrocytes (10^6^/μL)	8.7 ± 0.1 (*n* = 12)	8.5 ± 0.2 (*n* = 12)	10.3 ± 0.2 *** (*n* = 9)
Hemoglobin (g/dL)	14.7 ± 0.2 (*n* = 12)	15.6 ± 0.3 (*n* = 12)	18.9 ± 0.2 *** (*n* = 9)
Heart weight (g)	0.87 ± 0.1 (*n* = 6)	0.92 ± 0.1 (*n* = 8)	0.94 ± 0.08 (*n* = 6)

^1^ Evolution of body weight as % change from day 0 to day 14 after exposure, and blood parameters at day 14 are shown. Data are expressed as mean ± standard error (SEM) of 8–24 individual data and differences were obtained by repeated measures ANOVA; * *p* < 0.01; *** *p* < 0.001 vs. the control group (C) (one-way ANOVA).

## Data Availability

The data that support the findings of this study are available from the corresponding author upon request. The data are not publicly available due to pending upload to the repository of the University of Valladolid.
